# Parents and Children—Their True Relation

**Published:** 1883-07

**Authors:** Ben. H. Pratt


THE BISTOURY.

Vol. ~~XX.~~ XIX. ELMIRA, N. Y., JULY, 1883. No. 2.

### Contributions.

### PARENTS AND CHILDREN—THEIR TRUE RELATION.

BEN H. PRATT.

The family relation is a partnership.

There is never any lack of business in a well regulated family.

There is always enough to do to keep each member of the family employed at all times. Each must have his and her duties.

There can be no well idlers in a family, nor should there be masters and servants, commanders and obeyers, nor autocratic rule.

The relations of husband, wife and children are those of partners in any business in which the work is not done by employes but by the members of the firm.

The idea that the husband is the *master* of a household is one that obtains to an unfortunate extent the world over. He should be such in no sense whatever. He may, and should be the head—never the master.

It is an equally erroneous idea, too, that the wife is the *mistress* of the home, in the sense of an autocratic queen, whose word is law and to whom obedience is due because she *is* the mistress. Her province is a far different one from this.

That the children are all the natural servitors of the father and mother, bound to a blind obedience, without any rights or privileges except such as are granted by parental fiat, is another very unfortunate impression, and one coextensive with those before stated.

There should be, between the members of every family, a thorough understanding of the equality of each with each, just as there is between members of a business firm. That is, while there is a certain class of duties incumbent upon one and not required of another —none is master and none is servitor.

I do not wish to be understood as declaring that a parent has no right to command a child's service and compel its obedience; nor that a child is not bound to obey parental commands and yield obedience. To maintain such a theory would be subversive of all family government, and sap the very foundations of all domestic economy.

But, as in a business partnership, there is always a tacit understanding that, although each member of the corporation may be equal in every material particular to every other member, yet the general direction of the affairs of the firm must emanate from one only, so in the family should there be the same equality with the same singleness of general direction.

The commander is not of necessity the master, nor is the obeyer of necessity the servant, if it be understood that we command because it is our province to command, and obey because it is our province to obey. The distinction is plain, while the expression of the distinction may not be so. The primordial relation of the members of the family, each to each, implies command, obedience and service.

Now, in every well ordained family this is the first general point to be made clear to every individual in it that is capable of comprehending it: that command, though imperative, is not for the sake of commanding, and that service does not in any sense imply meniality; but that it is necessary, for the weal of the whole, that there be command, and that obedience to that command be equally implicit.

On each member of the family devolves a duty. The father has duties distinct, perhaps, from those of any member of his household. The mother's duties are equally defined and distinct. The duty of each child is no less a prescribed duty, or should be; and this brings me to the point I wish to impress, and the only point which my space here admits of my impressing, to wit: Each member in a family should have certain daily routine duties of his or her own.

I have not space to point out in detail, and scarcely can give even a general idea of the nature of the duties which each one in a family should be required to perform. The stations in life are so varied, the modes of living so different, the resources so wide asunder, the requirements of different families are so multiform, that it would be impossible for any one to direct intelligently or practically in this particular; but I can generalize sufficiently to convey the idea that I would impress.

It is not at all difficult to know what the duties of the father are. He is in general the purveyor. He gathers the means whereby the family shall be fed, clothed, taught and governed. His duties are oftener wholly without than wholly within the home. He is oftener the medium by which the family has knowledge of the outer world; the agent that comes between the source of supplies and the supplied. He is the protector of the home and of those within it, and the guardian of the interests of all those who are a charge upon him.

The duties of the mother of a family are more circumscribed in extent, perhaps, while they are fully as responsible, and frequently far more trying, than are those of the father. Her realm is within the home. It is her office to see that what is brought there be providently cared for and judiciously applied to the purpose designed; that the home be made the most comfortable, the most attractive, and the most refining spot it can attain to with such means as are provided her. Upon her often depends the happiness or misery of the household.

But it is of the children that I would especially speak. It is so common to find them the mere appendages of a family, and so little a part of it; so often either the mere pets or the absolute drudges; so often either not consulted at all, or consulted even to abject compliance with every whim. They are not made the equal partners of the home firm, each with his or her part of the business clearly defined and exacted, but either the employes, to be bidden about because employed, or the directors without wisdom, and accorded direction through a blind and mistaken parental affection.

The child that is brought up without regular, stated duties to perform, can never become a well-rounded, well-balanced man or woman. It does not matter so much what those duties are that we impose upon our children in the matter of co-operation with us in the routine affairs of the family, but these must be regular and constant. They may be trifling or important duties, dependent upon the age and capacity and bent of the child's talents, or genius, or disposition, or upon its strength. They should be daily, or at least periodical duties, and the performance of them should be insisted upon, even if they are not necessary to the well being or the happiness of the family. They must be done for the doing's sake.

It is common to find parents who, through a mistaken consideration for their children, cannot be led to impose upon them anything that may be irksome, or against which the child demurs, or from which it is continually begging to be excused because it interferes with play or visiting or other disposition of the time laid out by the child. It is often hard to deny a child an innocent diversion simply because it interferes with a stated duty or task. The parent may seem to be stern, or cruel, or lacking in natural affection, who will not remit for once, or twice, or frequently, an imposed duty. But it is demoralizing to do so, and cannot be done without injury to the forming habits of the child.

There is the other extreme found, in which the parent insists upon duties which are monopolists of all the time of the child, giving it no seasons of relaxation, no time of its own, making its life a continual term of labor. This is a custom as much to be avoided as that of exacting nothing in the way of duties, and allowing the child to be governed entirely by its inclinations and preferences. The poor are apt to expect that a child, arrived at a helpful age, shall bear as much of the family burdens as do the parents. The wealthy are apt to take the opposite course and require no service of their children except that which is voluntary, and even discouraging that because it may not be necessary.

Necessity may modify very materially the course parents pursue with their children in the direction of occupation. There must be exceptions to any rule that can be laid down in this matter, as circumstances can not always be controlled. But I would more particularly address that class which is not driven by dire necessity to lay burdens upon the children which they are not capable of bearing easily. Children should early be taught to help themselves in the daily routine of their lives; to get up without being bidden, and at a specified hour; to dress themselves without assistance at as early an age as possible; to get for themselves what they need rather than be allowed to call upon the parent or servant, or upon older brothers or sisters in the family.

There are hundreds of duties required in the family which may be performed by the smallest of the children. These need not be named. The observant father or mother may find little regularly recurring chores that the little one may just as well do as not, yet not because there is no one else to do it, nor yet because the doing of it is essential to the well being of the household, but merely that the child may have something to do regularly, and feel that it is an integral part of the working force of the family. Children do not seem to be of the family unless they are interested in it, and in no way can an interest be begotten in that child's mind without making it believe that it is doing a part of the family work. Its duties should be made to seem as important as possible.

Unless a child early becomes interested in the affairs of the family, and is to a certain extent taken into the confidence of the father and mother and elder members, it seems to grow up as an appendage to, rather than a part of the family. It can not, without being given to understand precisely how the family is situated, become reconciled to many things that it is called upon to do or to bear or to be denied. And nothing so quickly gives the child an insight into the inner affairs of the family as employment therein. It is a grievous error to let children grow up with mistaken ideas of their prospects and expectations. They can not know, without being told, why they may not do as their playmates do, dress as they dress, go where they go and have what they have.

There is but one mistake in family management greater than that of keeping children in ignorance of their station in life and the circumstances of their parents; and that is the concealment by the husband, from his wife, of the precise condition of his pecuniary circumstances. Such concealment is the cause of more domestic misery than any one other with which I am acquainted. And yet how few wives there are who know just how much or how little money their husbands are getting per annum. What a world of ruined men, despondent women, scattered households, marital infelicities and endless miseries might have been saved by the husband's making his wife the confidante of all his business transactions so far as they can affect the family relations for good or ill.

Next to perfect confidences between the father and the mother of a family comes the cultivation of a perfect understanding between parents and children; a thorough knowledge of the exact circumstances under which they are being reared. Unless this be done, there will be continually arising situations that either compromise or mortify. Children, unless tutored to it by confidential talks and explanations, cannot comprehend why they must do this, while others of their acquaintances are not compelled to do so; why they may not do another thing which some of their associates are doing; why all, in a word, are not upon the same social or financial level. Much perplexity and annoyance is saved for both children and parents if these matters are all understood and freely talked over in the family conclave. Deception should never enter that circle.

This matter thoroughly understood, and frequently and freely commented upon by all the family, old and young, makes many things clear to the child's comprehension that would otherwise remain a perpetual enigma, and a continual source of distrust between parents and children. Under such a system, special explanations for every occasion are not necessary, and many disappointments are avoided, and many of those schemes, the abandonment of which in the outset prevents anticipations that cannot be realized, are never sore-breeders in the child's heart. This, and occupation for all, and for each such occupation as is most congenial and most fitting, will smoothly carry any reasonably disposed family through those social and financial shoals that so beset the life of every household. Our pride must not stand in the way of our happiness. What we cannot afford to do, it is not right to do, and to do right is more consolatory than to be great.

Alas! what myriads of families are in constant misery over the mistaken views of parents in this matter of doing by their children better than they can afford to do, and thus conveying to the minds of these children false notions of the position in life they occupy. It is hard for the loving but straitened parent to deny his child a pleasure or a luxury which his child's playmate is enjoying without stint. It tempts him or her to make a sacrificial or a dishonest effort to keep pace with the luxuries of the more fortunate, and trust to the future to rectify the mistake. It is not only unwise to do this, but it is dangerous. It involves not only present unhappiness but frequently life-long misery, tutoring the child for a sphere which it must, in all probability, abandon for one more in accord with the circumstances which are sure to develop sooner or later. Then your children see how unwise you were, and your great affection for them will never be excuse sufficient, in their minds, for your short-sighted folly.

Parents should be honest with their children and with each other, above all things. Pretence is bad enough anywhere, but when it involves the present and future misery of offspring, it is simply despicable. Better far it is to tell your wife, your husband, your boy, your girl, the truth, and act upon it. Even poverty, with confiding and trustfulness, is better than the most brilliant riches with estrangements and misunderstandings. But what misery can compare with that which comes of poverty that is too proud to confess itself until it is too late to rectify the mistakes it has made. Every parent knows what can be afforded and what can not, and should be able to say no as promptly as yes, when no is the honest conviction of his or her heart. There is nowhere more constant demand for the exercise of judicial functions than in the parental relation. So show your love for your children that in all you do they will know that you do it for love of them. In all that you require them to do, have them understand that it is not service that you exact so much as it is co-operation with you in the weal-work of the family of which you and the children are equal component parts.

GUM TRAGACANTH.—One part of the powdered gum, mixed with fifty parts of water, and two parts of tincture of guaiacum added thereto must not turn blue within three hours; a blue color would show an admixture of gum-arabic.

